# High Glucose-Induced Oxidative Stress Mediates Apoptosis and Extracellular Matrix Metabolic Imbalances Possibly via p38 MAPK Activation in Rat Nucleus Pulposus Cells

**DOI:** 10.1155/2016/3765173

**Published:** 2016-08-22

**Authors:** Xiaofei Cheng, Bin Ni, Feng Zhang, Ying Hu, Jie Zhao

**Affiliations:** ^1^Shanghai Key Laboratory of Orthopaedic Implants, Department of Orthopaedic Surgery, Ninth People's Hospital, Shanghai Jiao Tong University School of Medicine, 639 Zhizaoju Road, Shanghai 200011, China; ^2^Department of Orthopedics, Changzheng Hospital, Second Military Medical University, Shanghai, China; ^3^Department of Toxicity Evaluation, Shanghai Municipal Center for Disease Control and Prevention, Shanghai, China

## Abstract

*Objectives*. To investigate whether high glucose-induced oxidative stress is implicated in apoptosis of rat nucleus pulposus cells (NPCs) and abnormal expression of critical genes involved in the metabolic balance of extracellular matrix (ECM).* Methods*. NPCs were cultured with various concentrations of glucose to detect cell viability and apoptosis. Cells cultured with high glucose (25 mM) were untreated or pretreated with N-acetylcysteine or a p38 MAPK inhibitor SB 202190. Reactive oxygen species (ROS) production was evaluated. Activation of p38 MAPK was measured by Western blot. The expression of ECM metabolism-related genes, including type II collagen, aggrecan, SRY-related high-mobility-group box 9 (Sox-9), matrix metalloproteinase 3 (MMP-3), and tissue inhibitor of metalloproteinase 1 (TIMP-1), was analyzed by semiquantitative RT-PCR.* Results*. High glucose reduced viability of NPCs and induced apoptosis. High glucose resulted in increased ROS generation and p38 MAPK activation. In addition, it negatively regulated the expression of type II collagen, aggrecan, Sox-9, and TIMP-1 and positively regulated MMP-3 expression. These results were changed by pretreatment with N-acetylcysteine or SB 202190.* Conclusions*. High glucose might promote apoptosis of NPCs, trigger ECM catabolic pathways, and inhibit its anabolic activities, possibly through a p38 MAPK-dependent oxidative stress mechanism.

## 1. Introduction

All forms of diabetes are preliminarily characterized by hyperglycemia. Although the molecular mechanisms that explain the relationship between diabetes and its complications have not been fully elucidated, accumulating pieces of evidence suggest that hyperglycemia-induced overproduction of reactive oxygen species (ROS) and subsequent oxidative stress act as a common pathway to the pathogenesis of late diabetic complications [1]. In addition to its ability to directly oxidize and damage DNA, proteins, and lipids, excessive production of ROS can function as signaling molecules to activate a number of cellular stress-sensitive pathways such as p38 mitogen-activated protein kinase (MAPK) [2], c-Jun N-terminal kinase, extracellular signal-regulated protein kinase [3], and NF-*κ*B pathway [4]. Activation of these pathways can alter the expression of genes that are involved in cell survival or death and cause cellular damage and apoptosis in hyperglycemia [5]. Intervertebral disc (IVD) degeneration is a major cause of low back pain [6], a condition that affects a significant proportion of the population [7]. The IVD is composed of the nucleus pulposus (NP) and the annulus fibrosus (AF). IVD degeneration is typically characterized by a loss of extracellular matrix (ECM). Apoptosis of nucleus pulposus cells (NPCs) plays a central role in the development of IVD degeneration [8]. Although the events leading to IVD degeneration are not well understood, there is ample evidence that oxidative stress and stress-induced signaling pathways play a role in the development of IVD degeneration [9–11]. Meanwhile, some investigations suggest that the progression of IVD degeneration is associated with diabetes and hyperglycemia [12–14]. However, to our knowledge, whether hyperglycemia-induced oxidative stress is implicated in the progression of IVD degeneration has not been investigated previously. In this study, we hypothesized that excessive ROS generation induced by elevations in glucose plays a key role in causing apoptosis of NPCs and abnormal expression of critical genes involved in the metabolic balance of ECM by its ability to activate the stress-sensitive p38 MAPK signaling pathway.

## 2. Materials and Methods

### 2.1. NPCs Isolation and Culture

The experimental protocol was approved by the local Institutional Animal Care and Use Committee and conformed to the Principles of Laboratory Animal Care. The IVDs were harvested from the lumbar spines of 12-week-old male Wistar rats immediately postmortem. The gel-like NP tissues were separated from the fibrous AF, washed with Hank's balanced salt solution (HBSS, Gibco, Grand Island, NY) and cut into small fragments. Minced tissues were digested with 0.4% pronase (Roche, Indianapolis, IN) for 30 minutes and then with 0.2% collagenase (Sigma, St. Louis, MO) for 3 hours. Tissue debris was removed by filtering through a cell strainer and isolated cells were rinsed twice with HBSS. The resulting cells were cultured with complete culture medium (DMEM (Gibco) containing 10% FBS (Gibco) and antibiotics) in a 37°C, 5% CO_2_ environment. The medium was changed every other day. After 10–14 days, the cells were harvested by brief exposure to 0.25% trypsin (Roche) and 1 mM ethylenediamine tetraacetic acid (Gibco) solution and replated into appropriate culture plates after washing with HBSS.

### 2.2. Determination of NPCs Viability in Various Concentrations of Glucose

The validity of NPCs was determined using the 3-(4,5-dimethylthiazol-2-yl)-2,5-diphenyltetrazolium bromide (MTT) assay. NPCs were cultured in 96-well plates (2 × 10^4^ cells per well) with complete culture medium for 12 hours, serum-starved for 12 hours, and then cultured in defined culture medium (DMEM containing 10% FBS and antibiotics) with various concentrations of glucose (0, 5, 15, or 25 mM, Sigma) for 12, 24, or 36 hours. NPCs in the culture medium were supplemented with MTT (0.5 mg/mL, Sigma) and incubated for 4 hours for viability assay. The blue formazan was dissolved in dimethyl sulfoxide and measured at 560 nm.

### 2.3. Treatments of NPCs

NPCs were serum-starved for 12 hours and then divided into four groups for subsequent experiments—control group: cells were cultured in defined medium for 24 hours; glucose group: cells were cultured in defined medium with various concentrations of glucose (5, 15, or 25 mM) for 24 hours; glucose + N-acetylcysteine (NAC) group: cells were pretreated with antioxidant NAC (10 mM, Sigma) for 12 hours before addition of 25 mM glucose; glucose + SB 202190 group: cells were pretreated with a specific p38 MAPK inhibitor SB 202190 (10 *μ*M, Sigma) for 12 hours before addition of 25 mM glucose.

### 2.4. Detection of Apoptosis

Apoptosis incidence was determined by flow cytometry analysis using the annexin V-FITC apoptosis detection kit (BD Biosciences, San Jose, CA). NPCs were harvested from each treatment group, collected together by centrifugation, and resuspended in 400 *μ*L of annexin binding buffer at a concentration of 1 × 10^6^ cells per mL. A 100 *μ*L sample of solution was treated with 5 *μ*L of annexin V-FITC and 5 *μ*L of propidium iodide at room temperature for 15 minutes, followed by the addition of 400 *μ*L of binding buffer. Staining cells were analyzed by a flow cytometer (EpicsAltra; Beckman Coulter, Fullerton, CA). Annexin V-FITC binding positive-staining cells were scored as apoptotic. Apoptotic cells were counted and represented as a percentage of the total cell count.

### 2.5. Measurement of Intracellular ROS

Production of intracellular ROS was evaluated by analyzing changes in fluorescence intensity resulting from the oxidation of the intracellular fluoroprobe 5′,6′-chloromethyl-2′,7′-dichlorodihydrofluorescein diacetate (CM-H2DCFDA, Molecular Probes, Eugene, OR). NPCs were incubated with 10 *μ*M CM-H2DCFDA for 30 minutes and the fluorescence intensity was measured with a fluorescent microplate reader at an excitation wavelength of 480 nm and an emission wavelength of 530 nm. CM-H2DCFDA-untreated NPCs were used to measure background fluorescence. The final fluorescent intensity was normalized to the respective protein content in NPCs used for each assay and then normalized to the fluorescent intensity of control cells.

### 2.6. Western Blot Analysis

After treatment, NPCs were immediately placed on ice and washed twice with ice-cold PBS. NPCs were lysed in Triton extraction buffer (PBS, 0.5% Triton X 100, 2 mM PMSF, and 0.02% NaN_3_) containing a protease inhibitor cocktail (Roche), 5 mM NaF, and 1 mM Na_3_VO_4_. The cell lysates were centrifuged and subjected to SDS-PAGE gels, followed by electroblotting onto polyvinylidene fluoride membranes. Membranes were probed with rabbit phospho-p38 MAPK polyclonal antibody (Cell Signaling Technology, Danvers, MA) or rabbit p38 MAPK polyclonal antibody (Cell Signaling Technology). Blots were visualized using enhanced chemiluminescence reagents (Amersham Biosciences, Buckinghamshire, UK). Densitometric analysis was conducted with Quantity One 4.4.0 software (Bio-Rad, Hercules, CA).

### 2.7. Analysis of Gene Expression by RT-PCR

Gene expression was analyzed by semiquantitative RT-PCR. Total RNA was isolated from NPCs after treatment using an RNeasy Midi Kit (QIAGEN, Hilden, Germany) according to the manufacturer's instructions. cDNA templates were prepared from 2 *μ*g of total RNA using oligo (dT) primers and SuperScript II reverse transcriptase (Life Technologies, Gaithersburg, MD). Specific cDNA were then amplified by PCR using the primers designed to assure specificity (Table 1). These genes were selected from four categories, including ECM components (type II collagen and aggrecan), anabolic factor (SRY-related high-mobility-group box 9, Sox-9), catabolic factor (matrix metalloproteinase 3, MMP-3), and anticatabolic factor (tissue inhibitor of metalloproteinase 1, TIMP-1). All results were normalized to the expression of glyceraldehyde phosphate dehydrogenase (GAPDH). PCR amplification from cDNA was performed with a thermocycler (GeneAmp PCR Systems 9700; Applied Biosystems, Foster City, CA) in a final volume of 50 *μ*L containing 1.5 mM MgCl_2_, 2.5 U TaKaRa Taq DNA polymerases (TaKaRa BIO, Shiga, Japan), and 0.3 *μ*M specific primers. The cycling conditions were initial denaturation at 95°C for 10 minutes, followed by 40 cycles at 95°C for 10 seconds, primer annealing at 60°C for 20 seconds, and elongation at 72°C for 15 seconds. All PCR products were determined by agarose gel electrophoresis with ethidium bromide staining and visualized by UV transillumination. The gel images were analyzed by densitometry using Melanie III software (GeneBio, Geneva, Switzerland). Data were normalized to the expression of GAPDH and control cells to calculate relative mRNA levels of each target gene.

### 2.8. Statistical Analyses

Data are expressed as means ± SEM. The evaluation of statistical differences among the groups was determined by one-way ANOVA followed by SNK test or by Kruskal-Wallis test followed by Nemenyi test using SPSS 16.0 software (SPSS Inc., Chicago, IL). Significance was defined by *P* values < 0.05.

## 3. Results

### 3.1. Cells Viability and Apoptosis

**Figure 1:**
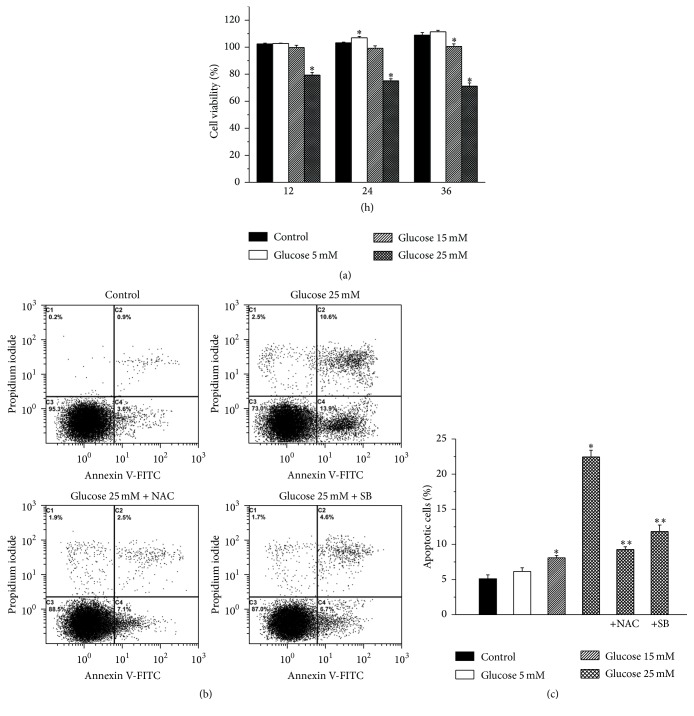
Viability and apoptosis of nucleus pulposus cells. (a) Viability evaluation of nucleus pulposus cells cultured with various concentrations of glucose after 12, 24, and 36 hours. (b) Representative sample of flow cytometry analysis of apoptosis using annexin V-FITC and propidium iodide staining. (c) The apoptosis rate of nucleus pulposus cells cultured with various concentrations of glucose. NAC: N-acetylcysteine, SB: SB 202190. Values are expressed as means ± SEM. ^*∗*^ *P* < 0.05 versus control; ^*∗∗*^ *P* < 0.05 versus 25 mM glucose group.

Figure 1(a) showed that when NPCs were maintained in a high glucose (25 mM) environment, there was a significant decrease in cell viability compared with control cells at each time point. In contrast, if the cells were exposed to 5 mM or 15 mM glucose, there was no significant difference in cell survival as compared to control cells, except for a higher value at a concentration of 5 mM at 24 hours and a lower value at a concentration of 15 mM at 36 hours. An increased rate of apoptosis was observed after 24-hour exposure of NPCs grown in a 15 mM or 25 mM concentration of glucose. No significant change in apoptosis rate was observed when NPCs were cultured with 5 mM glucose for 24 hours. The pretreatment with NAC or SB 202190 significantly decreased apoptosis rate of NPCs grown (Figures 1(b) and 1(c)). Therefore, we used a 25 mM concentration of glucose in subsequent experiments.

### 3.2. ROS Generation

**Figure 2:**
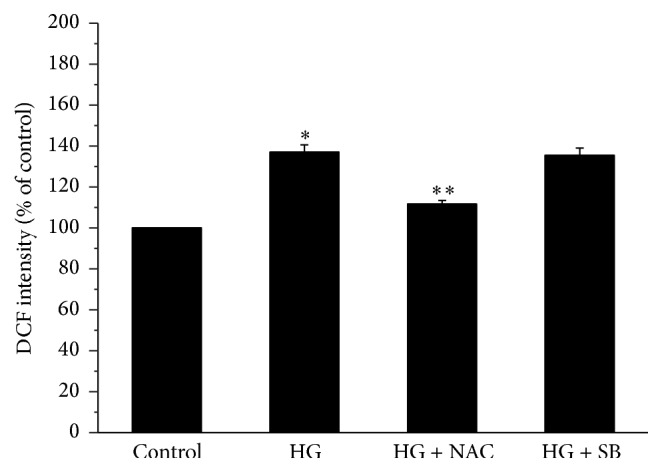
Effect of high glucose (HG, 25 mM) on reactive oxygen species (ROS) production in nucleus pulposus cells in the absence or presence of N-acetylcysteine (NAC) or SB 202190 (SB). The fluorescence dye 2′,7′-dichlorodihydrofluorescein (DCF) was used to monitor changes in ROS production. Values are expressed as means ± SEM. ^*∗*^ *P* < 0.05 versus control; ^*∗∗*^ *P* < 0.05 versus HG group.

NPCs treated with 25 mM glucose displayed significantly enhanced fluorescence intensity, which was significantly attenuated by the pretreatment with NAC but not SB 202190 (Figure 2).

### 3.3. Activation of p38 MAPK

**Figure 3:**
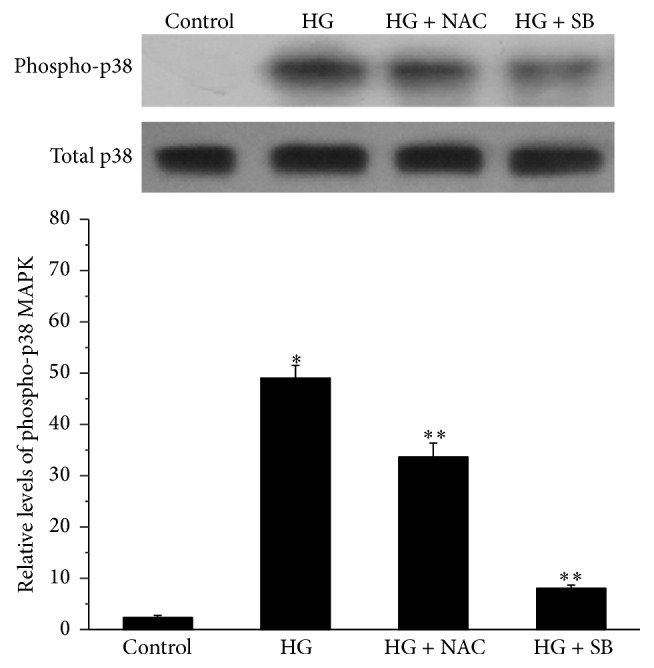
Representative Western blot and histogram showing p38 MAPK activation in nucleus pulposus cells treated with high glucose (HG, 25 mM) in the absence or presence of N-acetylcysteine (NAC) or SB 202190 (SB). Values are expressed as means ± SEM. ^*∗*^ *P* < 0.05 versus control; ^*∗∗*^ *P* < 0.05 versus HG group.

Exposure to a high concentration of glucose resulted in phosphorylation of p38 MAPK, while the levels of total p38 MAPK were not affected by the high glucose concentration. When pretreated with NAC or SB 202190, phosphorylated p38 MAPK levels of NPCs were lower than those without pretreatment (Figure 3).

### 3.4. Gene Expression

**Figure 4:**
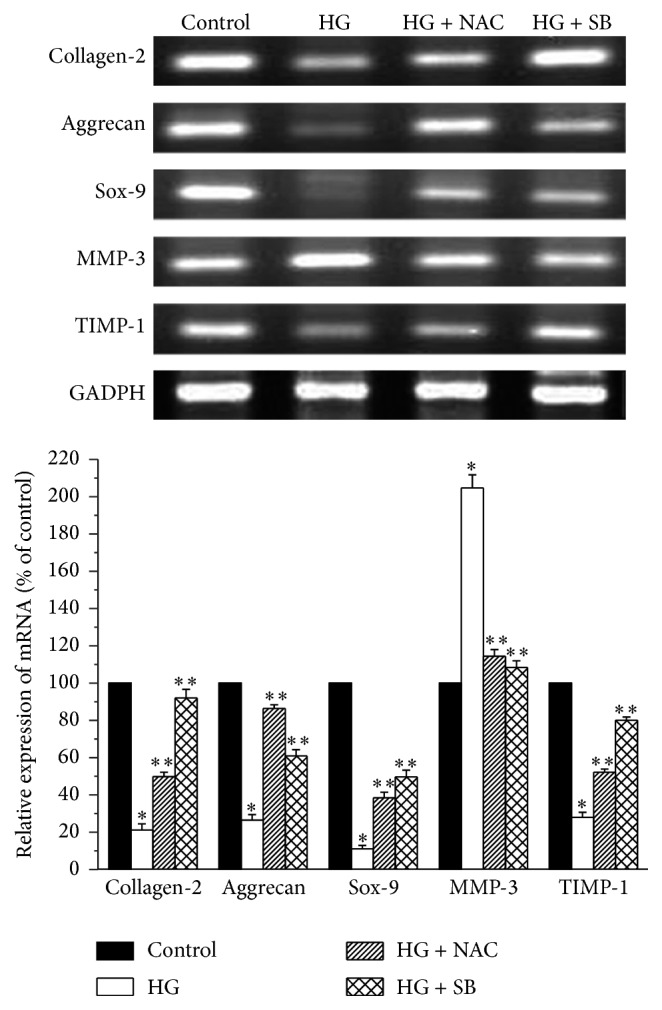
Representative RT-PCR and histogram showing the expression of mRNA in nucleus pulposus cells treated with high glucose (HG, 25 mM). The levels of type II collagen (collagen-2), aggrecan, SRY-related high-mobility-group box 9 (Sox-9), matrix metalloproteinase 3 (MMP-3), tissue inhibitor of metalloproteinase 1 (TIMP-1), and glyceraldehyde phosphate dehydrogenase (GAPDH) expression were quantified in the absence or presence of N-acetylcysteine (NAC) or SB 202190 (SB). Values are expressed as means ± SEM. ^*∗*^ *P* < 0.05 versus control; ^*∗∗*^ *P* < 0.05 versus HG group.

Figure 4 showed that NPCs under high glucose conditions expressed low levels of type II collagen, aggrecan, Sox-9, and TIMP-1 mRNA and high levels of MMP-3 mRNA compared with those under normal glucose conditions. When NPCs were pretreated with NAC or SB 202190, there appeared to be marked differences in the expression of all the transcripts.

## 4. Discussion

The present study showed that high glucose (25 mM) results in increased apoptosis of NPCs. The effect of glucose on NPCs was concentration-dependent because low glucose concentration (5 mM) did not increase apoptosis rate of NPCs. On the contrary, low glucose concentration increased cell viability at 24 hours, which might be attributed to more sufficient energy supply. Our experimental findings demonstrated increased formation of ROS in NPCs treated with high glucose. Excessive intracellular ROS generation in hyperglycemia is thought to occur via several well-studied mechanisms [1]. Increased formation of ROS results in a shift of the cellular redox balance and promotes enhanced intracellular oxidative stress in hyperglycemia [15]. Since it has been shown that hydrogen peroxide was able to induce apoptosis [16], many authors have reported that ROS could induce apoptosis in many different cell systems. In addition, there are numerous examples of the inhibition of apoptosis through antioxidative drugs or enzymes [17]. Our results showed that high glucose-induced oxidative stress caused a significant increase in the number of apoptotic NPCs, which was confirmed by the efficiency of the antioxidant treatment with NAC in inhibiting apoptosis. These results are consistent with previous findings that ROS can cause apoptosis of notochordal cells in the rat IVD and that inhibition of inappropriate or premature oxidative stress-induced apoptosis may delay the starting point of IVD degeneration [9]. In addition to the direct effect on apoptosis, oxidative stress has been shown to be relevant to accelerated cellular senescence in degenerate IVD, which can be used to explain the pathogenesis of IVD degeneration [18–20]. ROS-induced premature senescence has been observed in human IVD cells and insulin-like growth factor-1 has been proved to have potential to prevent or ameliorate senescence [10]. Gene expression and protein expression of caveolin-1 that is correlated with oxidative stress have been demonstrated within the IVD, supporting a role for oxidative stress in degenerative changes of the IVD [11].

Excessive ROS generation not only directly damages cells by oxidizing a variety of cellular macromolecules, but also indirectly damages cells by activating a variety of stress-sensitive intracellular signaling pathways [2–4]. Activation of these pathways results in the increased expression of numerous gene products that also cause cellular damage. Our experimental evidence substantiated that p38 MAPK activation mediated apoptosis of NPCs induced by high glucose and subsequent oxidative stress. Inhibition of p38 MAPK with SB 202190 significantly decreased the number of apoptotic cells. p38 MAPK is known as a stress-activated kinase and its activation occurs in response to hyperglycemia in a number of cell types, some of which are mediated by increased ROS production [21, 22] and could be restored by antioxidants [23]. In this study, increased ROS generation and p38 MAPK activation were both ablated by the pretreatment with the antioxidant NAC, while the pretreatment with SB 202190 did not inhibit the increased ROS generation, confirming that the p38 MAPK pathway is an oxidative stress-sensitive signaling system that can be activated by hyperglycemia and excessive ROS production in vitro. In IVD cells from various species, the p38 MAPK pathway participates in the cellular responses to different stimuli, such as inflammatory cytokines [24, 25], osmotic stress [26], and mechanical stress [27], resulting in cellular damage, senescence, and apoptosis. Inhibition of p38 MAPK in cytokine-activated IVD cells blunts the production of factors associated with IVD degeneration and may provide a therapeutic approach to slow the course of IVD degeneration [24, 25]. In addition, the p38 MAPK signaling pathway has been shown to play a key role in IVD matrix metabolism, as treatment with chemical inhibitors of p38 MAPK significantly counteracts the cytokine-induced decrease in ECM content, synthesis, and release [28].

It is important to note that high glucose state of NPCs appears to regulate the expression of critical mRNA transcripts involved in the metabolic balance of ECM. Aggrecan and type II collagen are major components of NP matrix [29]. We found that, at high glucose conditions, there was downregulation of type II collagen, aggrecan, and Sox-9 gene expression. Since Sox-9 regulates both type II collagen and aggrecan expression, the possibility existed that high glucose regulated the expression of Sox-9, and then this transcription factor influenced the expression of the two other genes [30].

MMP-3 and TIMP-1 have been reported to be correlated with IVD degeneration [8, 31]. Our results showing upregulation of MMP-3 and downregulation of TIMP-1 transcript, respectively, suggested that high glucose conferred degradative effects on NPCs, favoring ECM catabolism. The antidegradative effects of NAC and SB 202190 on high glucose-treated NPCs indicated that the regulation of these transcripts was probably mediated by oxidative stress-induced p38 MAPK activation. It has been reported that p38 MAPK is involved in the expression of these transcripts in many different cells systems [32–35]. Taken together, it was likely that high glucose negatively regulated the anabolic and anticatabolic gene expression of NP matrix and positively regulated the catabolic gene expression, resulting in a loss of water-binding capacity of ECM. However, we used p38 MAPK inhibitor but not siRNA molecules specifically designed for the loss of expression of all p38 MAPK isoforms and did not observe the effect of NAC and SB 202190 on all aspects tested in this study. In addition, we did not perform a quantitative gene expression analysis. Therefore, according to our data, p38 MAPK was just one of the possible mechanisms involved in the regulation of these transcripts. Some other mechanisms might also be implicated and need to be investigated in further study.

## 5. Conclusions

Our data demonstrated that the high concentration of glucose might promote apoptosis of NPCs, trigger ECM catabolic pathways, and inhibit its anabolic activities, possibly through a p38 MAPK-dependent oxidative stress mechanism.

## Figures and Tables

**Table 1:** Primers for RT-PCR.

	Forward	Reverse
Type II collagen	AAGGAGAACCTGGAGACATCAAG,	GTCACCACGGTCACCTCTG
Aggrecan	GCTACGGAGACAAGGATGAGTTC,	CGTAAAAGACCTCACCCTCCAT
SRY-related high-mobility-group box 9	GCTCCGACACCGAGAATACAC	TTGTCCTCTTCGCTCTCCTTCTT
Matrix metalloproteinase 3	AGCCAATGGAAATGAAAACTCTTC	CCAGTGGATAGGCTGAGCAAA
Tissue inhibitor of metalloproteinase 1	AGCAGAGCCTGCACCTGTGT	CCACAAACTTGGCCCTGATG
Glyceraldehyde phosphate dehydrogenase	GATGCTGGTGCCGAGTAC	GCTGAGATGATGACCCTTTTGG
